# Isothermal Microcalorimetry of Tumor Cells: Enhanced Thermogenesis by Metastatic Cells

**DOI:** 10.3389/fonc.2019.01430

**Published:** 2019-12-18

**Authors:** Douglas Lemos, Thaís Oliveira, Larissa Martins, Vitória Ramos de Azevedo, Mariana Figueiredo Rodrigues, Luisa Andrea Ketzer, Franklin David Rumjanek

**Affiliations:** ^1^Laboratório de Bioquímica e Biologia Molecular Do Câncer, Instituto de Bioquímica Médica Leopoldo de Meis, Universidade Federal Do Rio de Janeiro, Rio de Janeiro, Brazil; ^2^Núcleo Multidisciplinar de Pesquisa UFRJ-Xerém em Biologia (NUMPEX-Bio), Universidade Federal Do Rio de Janeiro, Duque de Caxias, Brazil

**Keywords:** thermogenesis, microcalorimetry, metastasis, UCP2, etomoxir, fatty acid oxidation

## Abstract

Tumor cells exhibit rewired metabolism. We carried out comparative analyses attempting to investigate whether metabolic reprograming could be measured by isothermal microcalorimetry. Intact metastatic cell lines of tongue cell carcinoma, human and murine melanoma, lung, and breast tumors consistently released more heat than non-metastatic cells or cells displaying lower metastatic potential. In tongue squamous carcinoma cells mitochondrial enriched extract reproduced the heat release pattern of intact cells. Cytochalasin D, an actin filament inhibitor, and suppression of metastasis marker Melanoma associated gene 10 (MAGEA10) decreased heat release. Uncoupling protein 2 was highly expressed in metastatic cells, but not in non-metastatic cells. Carnitine palmitoyl transferase-1 inhibitor, Etomoxir strongly inhibited heat release by metastatic cells, thus linking lipid metabolism to thermogenesis. We propose that heat release may be a quantifiable trait of the metastatic process.

## Introduction

Evidence has accumulated to show convincingly that the metabolism of tumor cells differs significantly from that of the majority of normal cells. Irrespective of the multiple alterations contributing to the so-called metabolic reprogramming, it is generally agreed that the major differences implicate the glycolytic and the tricarboxylic cycle pathways coupled to the oxidative phosphorylation (OXPHOS) system (1, 2). This is not surprising in view of the roles played by these pathways as the principal suppliers of ATP for tumor cells. In this context tumor cells are broadly classified according to the prevailing type of metabolism i.e., glycolytic or oxidative. The former derive ATP mainly from aerobic glycolysis, whereas the latter are able to recruit OXPHOS to fulfill their extra energy demands (3). However, classifying tumors as anaerobic or oxidative is not simple. The difficulties stem from the extensive and highly connected pathways, themselves being amenable to fine regulation at various levels. The precise classification of tumor cells according to the type of metabolism would require kinetic measurements of the individual key regulatory enzymes of glycolysis, pentose phosphate pathway, and OXPHOS, to name a few—plus all the relevant anaplerotic branches feeding into those cycles. To date, comprehensive biochemical models that take into consideration data from enzyme kinetics, metabolomic and fluxomic analysis and thus explain how the tumor cells apportion energy toward various processes are scarce. An explanation should take in to consideration proliferation, intravasation into blood and lymphatic vessels and migration and colonization of distant tissues as it occurs in metastasis. For these reasons sorting tumor cells as glycolytic or oxidative is still not consensual (4). As if this were not complicated enough there is growing evidence showing that many enzymes of the glycolytic pathway have dual roles, i.e., besides exerting their canonical metabolic functions they can also act as transcription factors (5). We reasoned that one way of simplifying this scenario would be to investigate the bioenergetics of tumor and metastatic cells by focusing on a parameter intimately associated to virtually all cellular events, as thermogenesis for instance. The central question raised in the present work was: energy wise what are the general features exhibited by cells undergoing metastasis? This was dealt with by carrying out isothermal microcalorimetry assays with intact cells bearing distinct metastatic potentials. The aim was to prospect whether there were thermogenic differences between the cell lines and to correlate those with certain metabolic pathways, as well as pinpointing the organelles responsible for the adaptive thermogenesis.

## Materials and Methods

### Cell Lines

Tongue squamous carcinoma cells (SCC-9, LN-1, and LN-2) were a kind gift by Dr. Michelle Agostini (6). Human melanoma cells WM983A and WM9838B and WM852 (7) were a kind gift by Dr. Michelle Botelho of Federal University of Rio de Janeiro. Murine melanoma cells 4C, 4C11–, and 4C11+ were a gift by Dr. Miriam Jaisiulionis (8). Human non-small-cell lung cancer cell lines A549 and NCI-H460 and human breast adenocarcinoma cells MCF-7 and MDA-MB-231 were acquired commercially. Metastatic potentials of the cells were determined elsewhere (6, 7) and obeyed the following hierarchy: tongue: LN-2 > LN-1 > SCC-9; murine melanoma: 4C11^+^ > 4C11^−^ > 4C; human melanoma: WM852 > WM9838B > WM983A; breast: MDA-MB-231 > MCF-7; lung: H460 > A549. Mycoplasma contamination was tested by PCR using primers described elsewhere (9).

### Monolayer Cell Cultures

Unless otherwise stated cells were grown in RPMI 1640 medium (Gibco) supplemented with (10%) fetal bovine serum (FBS; Gibco BRL) and antibiotic penicillin and streptomycin (LGC Biotechnology). Tongue squamous carcinoma cells and human melanoma were cultivated in DMEM **/**F-12 and DMEM, respectively, pH 7.2 supplemented with (10%) fetal bovine serum (FBS; Gibco BRL), and antibiotics penicillin and streptomycin (LGC Biotechnology) at 37°C in a humidified atmosphere of 5% CO_2_ in a Series 8000 WJ CO_2_ incubator (Thermo Scientific). Approximately 7 × 10^5^ cells were seeded in cell culture dish 100 × 20 mm (Sarstedt 83.3902) and grown to 90% confluence. Cells were then treated with 0.25% (W/V) trypsin (Sigma) solution containing 0.78 mM EDTA to become detached.

### Microspheroid Formation

Ninety-six U shaped well plates (Corning Costar 3799) were used. 24 h before platting the wells were layered with 1% agarose to favor microspheroid formation. 3 × 10^4^ cells/well were used for seeding. Human oral squamous carcinoma cells were incubated for 72 h under an atmosphere of 5% CO_2_ at 37°C in a CO_2_ incubator.

### Microspheroid Dissociation

Microspheroids were gently washed twice with phosphate buffered saline pH 7.4. After centrifugation at 400 g for 5 min, cells were treated with 0.125% (W/V) trypsin (Sigma) solution containing 0.78 mM EDTA for 5 min at 37°C. After incubation, medium containing fetal bovine serum was added to inactivate trypsin and cells were then gently dissociated by repeated cycles of aspiration with a 1 mL automatic pipette until the spheroids were visually undetected. Dissociated cells were then centrifuged at 400 g for 5 min.

### Cell Viability

Cell viability was assayed by the MTT method (10) and LDH release (11).

### RNA Extraction and cDNA Synthesis

Total RNA was isolated from cells using TRIzol reagent (Invitrogen) according to the manufacturer's instructions. Total RNA was quantified spectrophotometrically. 1 μg RNA was treated with 1 U of RNAse-free DNAse for 30 min at 37°C. Reactions were stopped by adding 1 μL of EDTA 20 mM and heating for 10 min at 65°C. cDNA synthesis was performed using the DNAse treated RNA using the High Capacity cDNA Reverse Transcription Kit from Applied Biosystems according to the manufacturer's instructions.

### Suppression of Human MAGEA10

Stable **s**pecific **s**uppression of MAGEA10 mRNA was achieved by transduction of lentiviral particles expressing oligonucleotides bearing a short hairpin structure as described elsewhere (12).

### Real Time PCR

Gene expression analysis was performed using 7500 Real Time PCR (Applied Biosystems) and power SYBR-GREEN PCR master MIX (Applied Biosystems). For this test primer pairs were synthesized based on GenBank sequences of mRNA. The comparative Ct method was used to measure changes in gene expression levels (13). Actin was used as an endogenous control.

### Microcalorimetry

Heat production was measured using an OMEGA Isothermal Titration Calorimeter VP-ITC from Microcal Inc. (Northampton, MA). The calorimeter sample chamber (1.8 ml) was filled with medium without FBS and the reference chamber was filled with Milli-Q water. After equilibration at 37°C, the reaction was started by injecting intact cells, or when indicated cell-free extracts into the sample chamber. The heat change was recorded at 5 min intervals during 35 min. The total volume of the cell suspensions injected in the sample chamber was 120 μL, and the cell count for each measurement was 1.2 × 10^5^. The heat change measured during the initial 5 min after cell injection was discarded in order to avoid artifacts such as heat of dilution. The results were expressed by columns which represented the total heat output after 35 min incubation. Although the raw data is conventionally expressed by negative values (exothermic heat dissipation), for the sake of clarity the results were plotted as positive values. In the ITC protocol used, the results were recorded after a single injection of cells. Heat release was not recorded during longer periods in order to avoid exposing the cells to hypoxic conditions. Oxygen consumption of the cells was independently monitored using high resolution oxygraphy with Oroboros 2K equipment and oxygen was still available in the chamber after 35 min of experiment (see Supplementary Figure 1). The heat production protocol was adapted from de Meis et al. (14). The data were plotted using the inbuilt software Origin 5. The concentrations of inhibitors used in the microcalorimetry experiments are specified in the legends of the figures. All treatments were performed immediately before the start of each experiment.

### Mitochondria Preparations

10^6^ cells were homogenized with 30 passes in a Potter-Elvehjem homogenizer in ice, in a buffer containing 10 mM Tris-HCl pH 7.4, 0.25 M sucrose, 20 mM NaF and 5 mM EDTA (cell lysis buffer). The homogenates were centrifuged for 5 min at 4°C at 1,000 g. The supernatant was collected and centrifuged for 15 min at 4°C at 10,000 g. The supernatant (cytosolic fraction) was saved and the pellet (mitochondrial fraction) was suspended with the cell lysis buffer. Protein concentration was assayed using the Bradford method. For the microcalorimetry assay, DMEM/F-12 medium without serum was added to both fractions, mitochondrial and cytosolic. For the calorimetry assays of mitochondrial and cytoplasmic preparations heat exchange was normalized for protein concentration.

### Western Blotting

Cell pellets were lysed in a Potter-Elvehjem homogenizer in ice during 3 min, in a buffer containing 10 mM Tris-HCl pH 7.4, 0.25 M sucrose, 20 mM NaF and 5 mM EDTA (cell lysis buffer). The homogenates were centrifuged for 5 min at 4°C at 1,000 g. The supernatant was collected and centrifuged for 15 min at 4°C at 10,000 g. The supernatant (cytosolic fraction) was saved and the pellet (mitochondrial fraction) was suspended with the cell lysis buffer. Protein concentration was assayed using the Bradford method. 40 μg of protein extracts were fractionated by standard 10% SDS-PAGE and transferred to nitrocellulose membranes by electro blotting in a buffer consisting of glycine 39 mM, Tris-base 48 mM, SDS 0.037% and methanol 20%. Proteins were detected using primary antibodies diluted in TBS, Tween 20 0.1% and BSA 5%. Mouse primary antibody against human UCP 2 was obtained from Abcam®. The secondary antibody was IRDye 800CW goat anti-mouse immunoglobulin. Bands were visualized in a Li-Cor Odyssey western blot imaging.

### Statistical Analysis

Statistical analysis was performed using GraphPad Prism 6 (GraphPad Software, inc.). The results are expressed as mean ± SEM values for n independent experiments. Comparisons between groups were done by one-way ANOVA and *a posteriori* Dunnett's test. When appropriate, unpaired Student's *t-*tests or Mann-Whitney's test were employed. Differences of *p* < 0.05 were considered to be significant.

## Results

### Metastatic Cells Release More Heat Than Non-metastatic Cells

**Figure 1:**
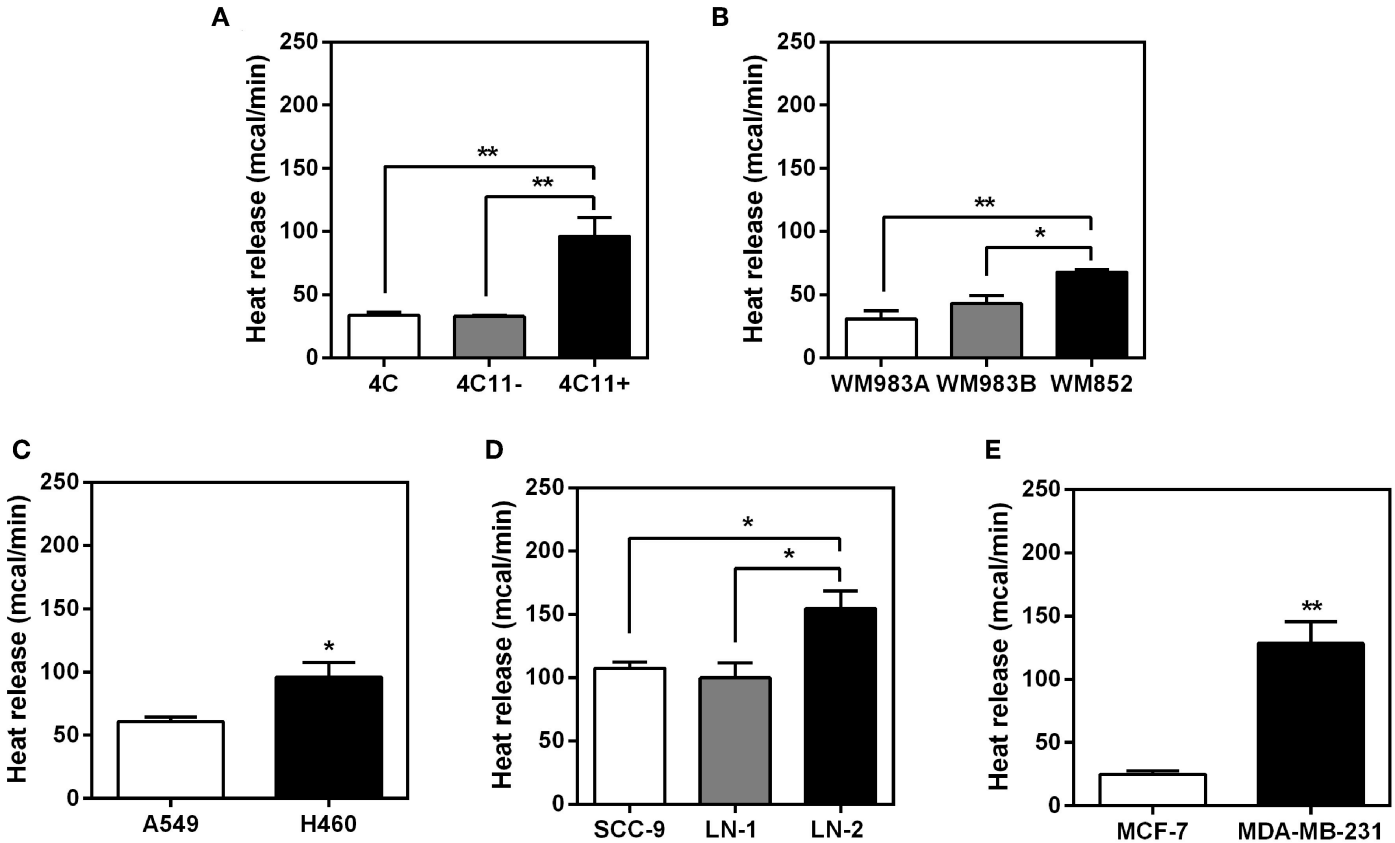
Heat release by different types of intact tumor cells.The bars represent the release of total heat of living cells in 35 min of experiment. Bars: white—non-metastatic tumor cells; gray - cells with intermediate metastatic potential; black - cells with high metastatic potential. **(A)** Murine melanoma cells 4C, 4C11− and 4C11+; **(B)** human melanoma cells WM983A, WM983B and WM852; **(C)** human non-small-cell lung adenocarcinoma cells A549 and H460; **(D)** human oral squamous carcinoma cells SCC-9, LN-1 and LN-2; **(E)** human breast cancer cells MCF-7 and MDA-MB-231. Values were expressed as mean ± SEM. *p < 0.05; **p < 0.01.

Intact cells from murine (4C, 4C11− and 4C11+) and human melanoma (WM983A, WM983B and WM852), lung (A549 and NCI-H460), tongue (SCC-9, LN-1 and LN-2) and breast (MCF-7 and MDA-MB-231) were used for the microcalorimetry assay. The results are shown in Figures 1A–E. Although individually each type of tumor cell displayed different maxima for heat release, in all cases the cells with the highest metastatic potential (4C11+, WM582, H460, LN-2, and MDA-MB-231) were consistently those displaying the highest absolute values of heat release. The total heat output reflected higher rates of heat release as shown in Supplementary Figure 2. These results show that heat release by the different cell lines as measured at 5 min intervals was constant over time although displaying clearly distinct slopes. The cells were kept under oxygen during the experiments as shown in Supplementary Figure 1.

The results shown in Figure 1 indicate that the positive correlation between the metastatic potential and heat release could be extended to several types of tumors (human or murine) with the same parental matrix or not. Whilst additional stable tumor cell lines exhibiting gradients of metastatic potential could have been added to the present list the authors believe that in this initial study a pattern can already be discerned that could be eventually generalized. For the remaining experiments described here only the human SCC tongue carcinoma cells were used. This decision was justified by the fact that with the exception of the murine melanoma cells, all other cell lines were derived from different parental matrixes (WM983B was derived from WM983A, but not WM852). Likewise for the human breast and lung cancer cells display different phylogenies. For example, MCF-7 cells are classified as luminal A, they contain estrogen and progesterone receptors and are considered as p53 wild-type. In contrast, the highly invasive MDA-MB-231 cells are classified as claudin-low (claudins are major integral membrane proteins of tight junctions), triple negative (ER^−^, PR^−^, and HER2^−^) and bear mutations on p53 (15), i.e., the two cell lines constitute altogether different cell types bearing different traits. Thus, for the sake of validating the comparative analysis of parameters relating to the functional aspects associated to the transition to metastasis along the same cell line, the subsequent experiments were conducted exclusively with the tongue squamous carcinoma cells (LN-1 and LN-2) since both were derived from SCC-9 cells after successive rounds of inoculation and recovery from lymph nodes (6). In attempt to mimic tumor organization *in vivo*, experiments were also conducted with 3D culture of tongue squamous carcinoma cells. Supplementary Figure 3 shows that the pattern of higher heat release in the metastatic cells LN-2 is maintained when compared to the heat released by the non-metastatic cells SCC-9. In LN-1 there was an increase in heat release when grown in spheroid.

### Suppression of MAGEA10 Reduces Heat Release by Tumor Cells

**Figure 2:**
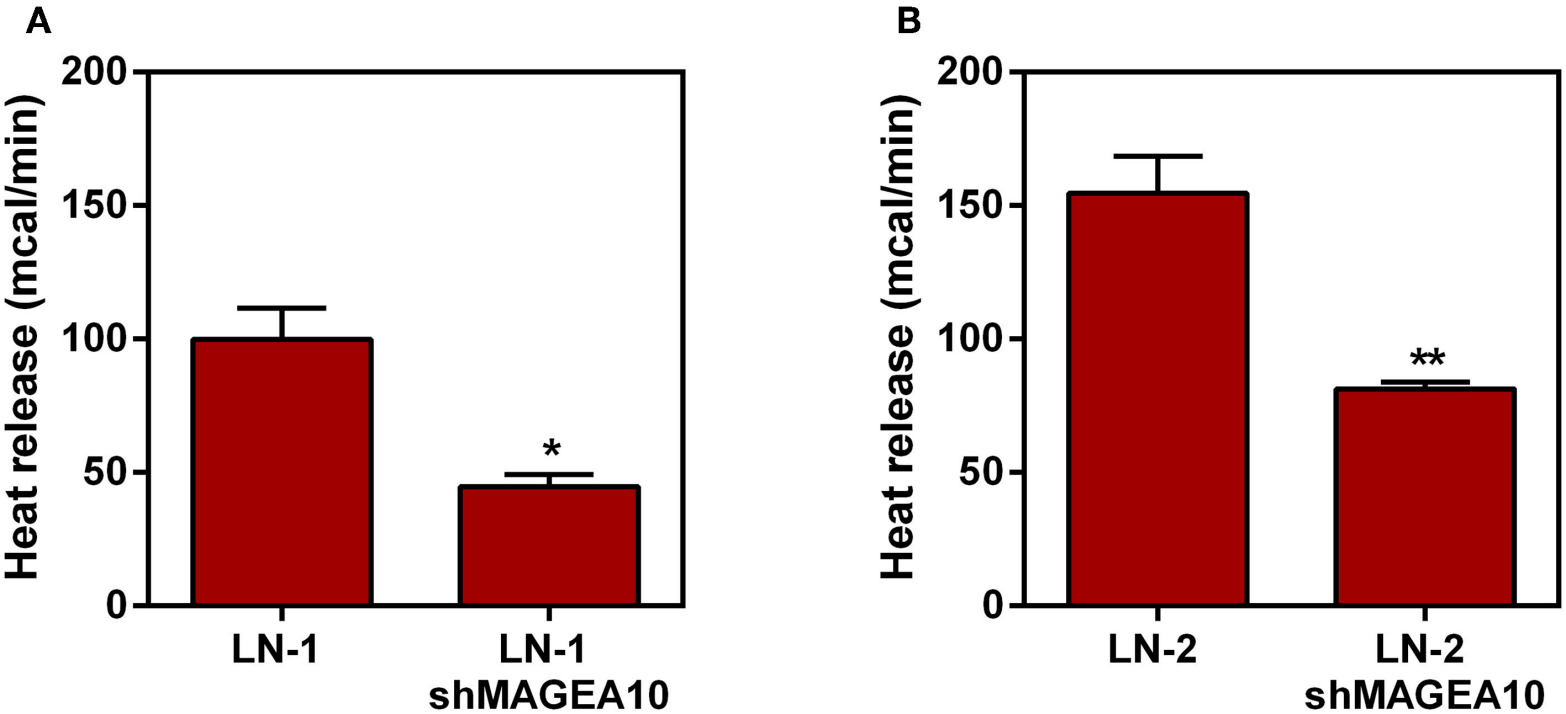
Heat release by human oral squamous carcinoma cells LN-1 and LN-2 suppressed for MAGEA-10 expression (shMAGEA10). The bars represent the release of total heat of living cells in 35 min of experiment. **(A)** LN-1 and LN-1 shMAGEA10 cells; **(B)** LN-2 and LN-2 shMAGEA10 cells. Values were expressed as mean ± SEM. *p < 0.05; **p < 0.01.

**Figure 3:**
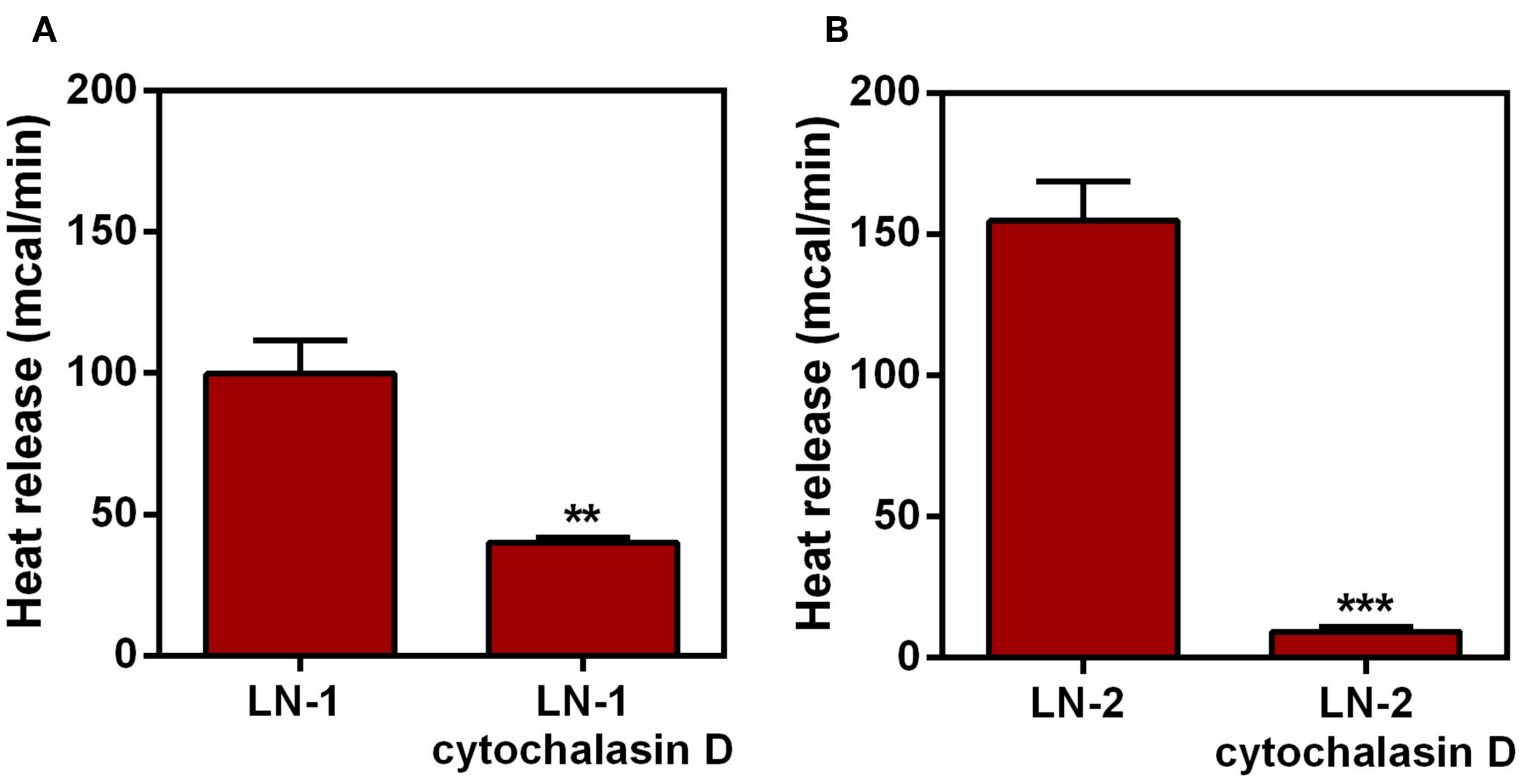
Effect of cytochalasin D on heat release by human oral squamous carcinoma cells LN-1 and LN-2. The bars represent the release of total heat of living cells in 35 min of experiment. **(A)** Heat release by LN-1 cells untreated and treated with cytochalasin D 2 mg/mL; **(B)** heat release by LN-2 cells untreated and treated with cytochalasin D 2 mg/mL. Values were expressed as mean ± SEM. **p < 0.01; ***p < 0.001.

Thus, we tested next whether the thermogenic differences observed in Figure 1 could be affected by the suppression of a protein that knowingly mediates adhesive properties of the LN cells. In a previous publication we have demonstrated that protein MAGEA10, which is highly expressed in metastatic cells, was involved in both, adhesion and motility of LN cells (12). Therefore, we carried out microcalorimetry experiments with LN cells stably suppressed for MAGEA10. The results are shown in Figures 2A,B. In both cell lines, LN-1 and LN-2, suppression of MAGEA10 (shMAGEA10) led to a significant reduction in heat release (Figures 2A,B). Although the reduction was more pronounced in LN-2 cells these were still more thermogenic than shMAGEA10 LN-1 cells (compare Figures 2A,B) a result which is compatible with the data shown in Figure 1. Also, suppression of MAGEA10 in both cell lines yields lower heat release than in untreated control SCC-9 cells. The differences in calorimetry measured between LN-1 and shMAGEA10 LN-1 and LN-2 and shMAGEA10 were ~60 mcal and 75 mcal, respectively, measured after 35 min. It is known that the movement of cells is driven by the continuous polymerization and reorganization of actin cytoskeleton (16). Following the same reasoning, we measured the heat release of metastatic cells (LN-1 and LN-2) in the presence of cytochalasin D, an actin polymerization inhibitor. As shown in Figure 3, cytochalasin D reduced heat release in both cells and more markedly in LN-2 (Figure 3B). Together, the results shown in Figures 2, 3 suggest that a link exists between proteins involved in adhesion and motility and the thermogenic behavior of LN cells.

### RNA and Protein Expression of UCP2 by Tumor Cells

**Figure 4:**
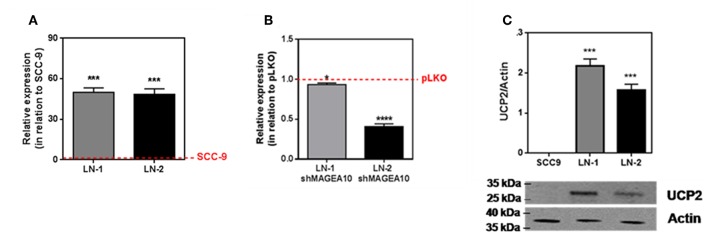
UCP2 mRNA and protein expression in human oral squamous carcinoma cells SCC-9, LN-1 and LN-2. **(A)** UCP2 mRNA levels in LN-1 and LN-2 cells relative to SCC-9 cells; **(B)** mRNA expression levels of UCP2 in cells supressed relative to pLKO. SCC-9 and pLKO (red line), LN-1 (gray bar) and LN-2 (black bar). **(C)** Protein levels of UCP2 in cells grown as monolayers quantified from the western blot shown in the inset. The values were expressed in relation to the red dashed lines. Values were expressed as mean ± SEM. *p < 0.05; ***p < 0.001; ****p < 0.0001.

An uncoupled protein (UCP) is a mitochondrial inner membrane protein that can dissipate energy in the form of heat during proton translocation (17). Nevertheless, to investigate this possibility we carried out experiments measuring the expression of uncoupling protein 2 (UCP2) by these cell lines. The results are shown in Figures 4A–C. UCP2 expression of LN-1 and LN-2 cells was much higher than SCC-9 cells (red dashed lines). Although UCP2 expression as shown by mRNA contents did not vary significantly when LN-1 and LN-2 cells were compared, the western blots shown in Figure 4C indicate that in LN-1 cells UCP2 protein was more abundant. A higher amount of translated protein doesn't necessarily mean higher activity. Additionally, Supplementary Figure 3 shows that UCP2 expression is much higher in 3D cultures than in monolayers a result that reinforces the idea that the spatial geometry of the cultures has a considerable effect on the protein transcription. The results in Figure 4B show that in LN-1 shMAGEA10 cells, expression of UCP2 is significantly different from that of control pLKO cells containing a scrambled insert (red dashed line). In contrast, in LN-2 shMAGEA10 cells, there was a reduction of more than 50% in the mRNA expression of UCP2, again corroborating the results shown in Figures 2, 3 and reinforcing the hypothesis that adhesive/motility properties of LN-2 cells may be connected to heat dissipation.

### Effect of Genipin on Heat Release by Tumor Cells

**Figure 5:**
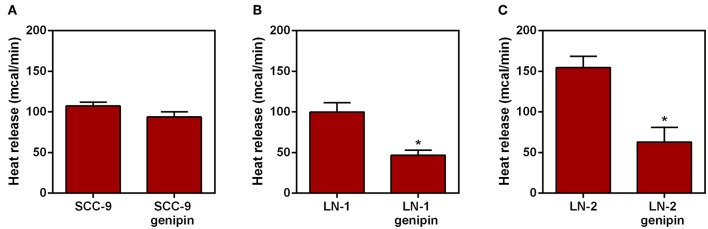
Effect of genipin on the heat release by human oral squamous carcinoma cells SCC-9, LN-1 and LN-2 cells.The bars represent the release of total heat of living cells in 35 min of experiment. **(A)** Heat release by SCC-9 cells untreated and treated with 50 μM of genipin; **(B)** heat release by LN-1 cells untreated and treated with 50 μM of genipin; **(C)** heat release by LN-2 cells untreated and treated with 50 μM of genipin. Values were expressed as mean ± SEM. *p < 0.05.

Additional evidence of the contribution of UCP2 to overall heat generation was obtained from experiments in which genipin was incubated with the intact cells followed by the microcalorimetry assay. Genipin is an aglycone that is readily incorporated by cells and acts as an inhibitor of mitochondrial enzyme uncoupling protein 2 (18, 19). The results are shown in Figures 5A–C. Whereas, genipin treatment did not significantly affect SCC-9 cells (Figure 5A), in both LN-1 and LN-2 cells, genipin promoted a clear inhibitory effect on heat release, particularly by LN-2 cells. The fact that genipin has no apparent effect on SCC-9 cells may be relevant to the result shown in Figure 4C which demonstrated that these cells do not synthesize UCP2. Thus, UCP2 mediated uncoupling may be a feature of metastatic cells. The differences in calorimetric measurements between treated and untreated LN-1 cells were 60 mcal (Figure 5B) and 90 mcal for LN-2 cells (Figure 5C) measured after 35 min. Taken at face value, the results in Figure 5 indicate that UCP2 has a role in the process of thermogenesis in metastatic cells.

### Effect of Oligomycin on Heat Release by Tumor Cells

**Figure 6:**
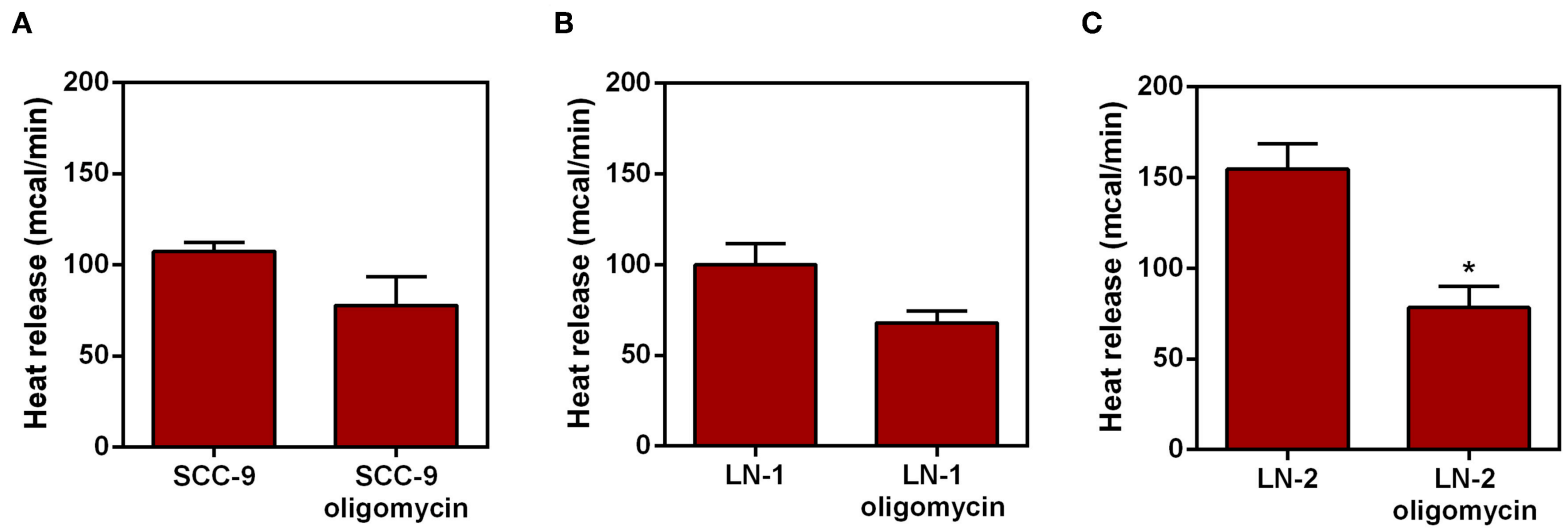
Effect of oligomycin on heat release by human oral squamous carcinoma cells SCC-9, LN-1 and LN-2. The bars represent the release of total heat of living cells in 35 min of experiment. **(A)** Heat release by SCC-9 cells untreated and treated with oligomycin 2 μg/mL; **(B)** heat release by LN-1 cells untreated and treated with oligomycin 2 μg/mL. **(C)** Heat release by LN-2 cells untreated and treated with oligomycin 2 μg/mL. Values were expressed as mean ± SEM. *p < 0.05.

The ATP synthase is a mitochondrial enzyme localized in the inner membrane that translocates protons (like UCP2). This proton translocation allows ATP synthesis from ADP (20). To verify whether the heat release observed in UCP2 modulation is related to proton translocation experiments were performed in which the intact cells were treated with oligomycin (2 μg/mL), a classical inhibitor of mitochondrial ATP synthase (21). The results are shown in Figure 6. LN-1 cells have a comparatively smaller inhibitory effect when compared to oligomycin treated LN-2 cells. Only LN-2 has significant reduction in heat release when treated with oligomycin (Figure 6C). The approximate difference between oligomycin treated samples and control were 25 mcal for SCC-9 control vs. oligomycin treatment, 40 mcal for LN-1 control vs. oligomycin treatment and 80 mcal for LN-2 control and oligomycin treatment, measured after 35 min. Oligomycin dependent reduction of thermogenesis in LN-2 cells is due to the binding of the antibiotic to the F_O_ portion of the mitochondrial F_O_/F1 ATPase. Prevention of proton flow back into the mitochondrial matrix may have thus reduced heat release. Conversely, in untreated LN-2 cells (Figure 6C) gradient derived proton flow into the matrix was unimpeded and thus promoted a higher heat release.

### Mitochondrial Enriched Extract From Metastatic Cells Are More Thermogenic Than Mitochondrial Extract From Non-metastatic Cells

**Figure 7:**
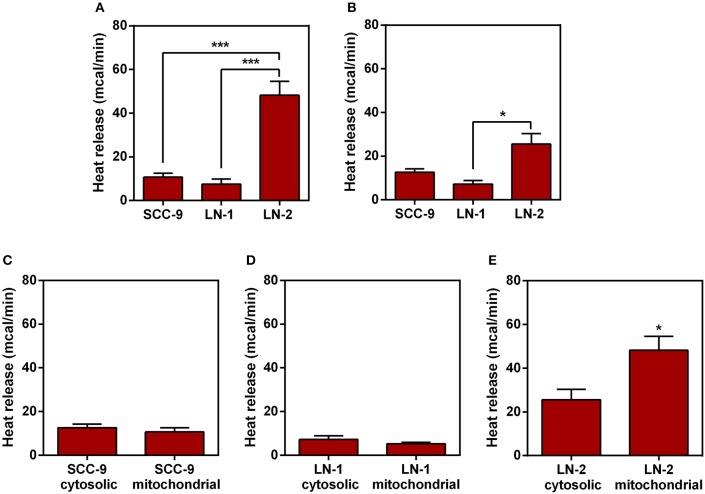
Comparison of heat release by protein extracts enriched with mitochondrial or cytoplasmic fractions.The protein extracts were obtained from human oral squamous carcinoma cells SCC-9, LN-1 and LN-2. The bars represent the release of total heat of protein extracts in 35 min of experiment. **(A)** Protein extracts enriched with mitochondrial fraction of SCC-9, LN-1 and LN-2; **(B)** protein extracts enriched with cytoplasmic fraction of SCC-9, LN-1 and LN-2; **(C)** protein extracts enriched with mitochondrial and cytoplasmic fractions of SCC-9 cells; **(D)** protein extracts enriched with mitochondrial and cytoplasmic fractions of LN-1 cells; **(E)** protein extracts enriched with mitochondrial and cytoplasmic fractions of LN-2 cells. Values were expressed as mean ± SEM. *p < 0.05; ***p < 0.001.

Next we investigated which organelles were involved in the thermogenesis of LN cells. Assuming that both UCP2 and ATP synthase are mitochondrial proteins and that inhibit their inhibition could cause a significant reduction on heat release by the metastatic cells LNs (Figures 5, 6) it is reasonable to assume that mitochondria may play a role in view of their central function in cell energy conversion. The next experiments were conducted with mitochondrial enriched and cytosolic extracts obtained by differential centrifugation. The results are shown in Figure 7. In Figure 7A, it can be seen that as far as LN cells are concerned the same heat release pattern observed in Figure 1 is reproduced, i.e., isolated mitochondria from the cells with the highest metastatic potential (LN-2) were also found to be the most thermogenic. In contrast, the results in Figure 7B showed that cytosolic extracts exhibited considerably less heat release than the mitochondrial extracts. Nevertheless, LN-2 cytosolic extracts were still more thermogenic than SCC-9 and LN-1 extracts. The relatively small difference observed in Figure 7B could be due to exothermic interactions occurring in the cytosol that are exclusive to the LN-2 cells. The LN-2 cytosolic protein network may be more complex and such interactions may reflect this high heat release. Figures 7C,D show that there were no differences in heat release between SCC-9 mitochondrial and cytosolic extracts, as well as between LN-1 mitochondrial and cytosolic extracts. The highest difference was observed when LN-2 mitochondrial and cytosolic extracts were compared (Figure 7E). The results in Figure 7 indicate that mitochondria could have been partially responsible for the thermogenic behavior of LN-2 cells.

### Effect of Etomoxir on Heat Release by Tumor Cells

**Figure 8:**
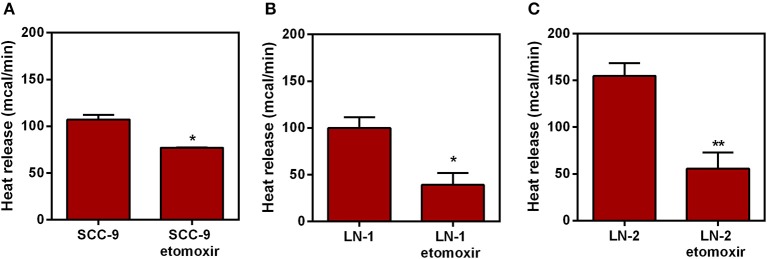
Effect of etomoxir on the heat release by human oral squamous carcinoma cells SCC-9, LN-1 and LN-2 cells. The bars represent the release of total heat of living cells in 35 min of experiment. **(A)** Heat release by SCC-9 cells untreated and treated with 300 μM of etomoxir; **(B)** Heat release by LN-1 cells untreated and treated with 300 μM of etomoxir. **(C)** Heat release by LN-2 cells untreated and treated with 300 μM of etomoxir. Values were expressed as mean ± SEM. *p < 0.05; **p < 0.01.

A link between UCP2 and fatty acid catabolism and transport has already been suggested (19). Prompted by this we then tested whether etomoxir, an inhibitor of the enzyme carnitine palmitoyl transferase-1 (CPT1), had any effect on the heat release by SCC-9 and LN-2 cells. The results are shown in Figure 8. Whereas, 300 μM etomoxir discreetly affected heat release by SCC-9 and LN-1cells (Figures 8A,B), the effect of the inhibitor on LN-2 cells was quite pronounced (Figure 8C). Etomoxir produced a reduction of ~110 mcal, a result which not only confirms that mitochondria are accessory to the thermogenic profile of metastatic cells, but also that this involves the participation of fatty acid oxidation on the energy metabolism of LN-2 cells. Reduced thermogenesis caused by 300 μM etomoxir could not be attributed to harmful effects on the cells, since neither LDH release nor the MTT viability assays (Supplementary Figures 4A,B) indicated cytotoxicity.

## Discussion

Isothermal titration calorimetry is a powerful and versatile technique that has been used extensively in chemistry and biology to measure thermodynamic parameters such as enthalpy, Gibbs free energy and binding affinities in chemical reactions and enzyme kinetics. Albeit not so numerous, the applications of ITC have gone beyond binary ligand reactions and also included the study of whole living cells. Thus, microcalorimetry studies have been applied to microorganisms (22–24) and to cells and tissues slices (25). When studying the interactions of biomolecules in solution the titration relies mainly on multiple injections of the samples, whereas with whole cells, single injections may be the method of choice (26). This was the approach utilized here.

Our results (Figure 1) showed that in several types of cancer a direct correlation existed between malignancy and total heat released. This observation supports the proposal that tumor cells do indeed display a reprogrammed metabolism and that metastasis might resort to metabolic pathways that supply extra energy to enable processes such as increased motility and invasiveness. The results described here obtained with cell suspensions are in agreement with the pioneering work of Kallerhoff et al. (27) who measured heat release of tissue samples from the human urogenital tracts, including prostate, bladder, kidney and testicular tissue. Kallerhoff et al. showed that it was possible to differentiate normal from tumor cells, although they could only speculate that the difference found may have been attributable to a higher metabolic activity.

As we observed this increase in heat release exclusively in metastatic cell lines among the different tissues used (Figure 1), we decided to investigate what would be the interference, an increase of enthalpy, modulating MAGEA10, a protein closely related to the metastatic characteristics of tongue squamous carcinoma cells as shown in a paper by our group (12). Silencing this protein promotes a significant reduction in heat release (Figure 2), particularly in LN-2 cells. In addition to adhesion/motility, a contribution from MAGEA10 proteins and other members of the MAGE family could be selected. For example, MAGE proteins are known to be assembled with E3 RING ubiquitin alloys to form MAGE-RING alloys (MRLs) that function in many cell lines, including tumor cell proliferation (28) and total heat production. As shown in a previous paper (12), by silencing MAGEA10 in LN-1 and LN-2 cells, we observed a reduction as measured by migration and invasion. Indeed, by inhibiting the polymerization of the actin with cytochalasin D, we observed a smaller heat release, especially in the more aggressive cell line (Figure 3). It has been previously shown that cytochalasin D effect, on cell migration, can be an antitumor mechanism (29). From this, we can infer that the low heat release observed in the LN-2 cell line, both in MAGEA10 silencing and cytochalasin D treatment, may be linked to the loss of metastatic characteristics such as migration and invasion (12, 30, 31).

In an attempt to investigate the heat source, we analyzed the expression of uncoupling proteins in tongue squamous cancer cell lines. The family of UCPs, mainly UCP2 and UCP3, is known to have a direct relationship with thermogenic signals from recurrent biochemical reactions within mitochondria, such as fatty acid oxidation (32, 33). Thus, in addition to the fact that UCP2 has a different expression in different tissues, it has been implicated as an enhancer of endothelial cell resistance to oxidative stress (34). As well as having a role in metabolic reprogramming in skin epidermal cells (35). Taken together, these results reinforce the correlation between UCP2 and metastasis (36). Our data shown that LN-1 and LN-2 cells have high UCP2 expression when compared to SCC-9 (Figures 4A–C). Furthermore, the silencing of MAGEA10 led to a significant reduction in UCP2 expression in the most metastatic line (Figure 4B). In Figures 1, 2, we observe that the increase in heat release is accompanied by a high expression of UCP2 in LN-2. Additionally, the reduction in thermogenesis, when LN-2 is silenced to MAGEA10, is accompanied by a low expression in the UCP2 gene in this cell line. These results suggest that this uncoupling protein plays an important role in the thermogenic levels of metastatic cells of tongue squamous carcinoma. This interpretation was consistent with the results shown in Figure 5, which show that UCP2 inhibitor genipin significantly reduced the heat released by LN-1 and LN-2 cells, but not by SCC-9 cells. Notwithstanding this observation, it should be mentioned that genipin is known to have other effects than UCP2 inhibition. This includes anti-proliferative actions on tumor cells (37). Genipin has also been shown to be a water soluble crosslinking agent (38). Given the last one property of genipin, it would not be surprising to observe a reduction in heat release promoted by the inhibitor, since interactions between polymers are generally exothermic. Since no genipin effect was observed in SCC-9, a cell line that not expressing UCP2 (Figure 4C). It is plausible that the reduction in heat release observed in LN-1 and LN-2 does not occur due to the possible non-specific effects of genipin.

Considering the possible interference of UCP2 in thetermogenic mechanism of metastatic cells, we directed our investigation in order to find the major source of this heat released. As UCP2 is a mitochondrial uncoupling protein, we evaluated the heat released by this organelle. The results shown in Figure 7 confirmed that preparations cell-free extracts enriched in mitochondria were more thermogenic than cytosolic extracts and that mitochondria obtained from the more aggressive LN-2 cells were also more thermogenic than mitochondria from LN-1 and SCC-9 cells. Whilst those results substantiated the idea that mitochondria may be responsible, at least partially, for the heat output of the metastatic cells, it must be mentioned that the heat released by the isolated organelles may differ from that measured in intact cells. Ideally the comparison should be conducted by measuring the absolute thermal contribution of mitochondria within the cellular milieu. Thus, our data point to a possible mitochondrial contribution in metastatic cell thermogenesis. Although there is little information, thus contributing to cell migration, about mitochondrial regulation of migration, it is known that this organelle can interact closely with the endoplasmic reticulum to perform Ca^2+^ signaling, aiding in the mechanism of metastatic cell migration (39, 40). It has been reported that the AMPK pathway may participate in the mechanical transduction of motility events in breast cancer cells such as MDA-MB-231 (41). Thus, we can suggest that the high release of heat energy by mitochondria may help the migratory/invasive potential of the most aggressive cell lines.

Therefore, aware of the high heat release in mitochondrial extracts, and the high regulation of UCP2 through more or less intense thermogenic signals from fatty acid oxidation in mitochondria (32, 33), we analyzed the interference of β-oxidation inhibition on the global heat release of cell lines. Our data showed that when treated with etomoxir, the cell lines were less thermogenic (Figure 8), with the most dramatic reduction in LN-2. By further investigating the metabolism of tongue squamous carcinoma cells, the results presented here extend our previous work (42) and reinforce the idea that metastatic cells extract the excess energy from mitochondrial pathways, particularly by channeling ATP from fatty acid oxidation. Similar results were obtained by our group using melanoma (43) and human breast cancer cells (44). It is conceivable that the role of lipid metabolism in metastasis involves not only energy production but also that of a building block supplier for membrane biogenesis. Metabolic pathways involving fatty acid can also generate signaling lipids (45, 46). Our own results, using high-resolution oxygen, confirmed the implication of lipid metabolism in metastasis. A comparative analysis of mouse melanoma cells exhibiting different degrees of metastatic potential showed that fatty acid oxidation was significantly increased in the more aggressive cell lines (43). In addition, metabolomic analysis of LN-2 cells showed that they were able to accumulate among other metabolites, malonate, methyl malonic acid, n-acetyl, and unsaturated fatty acids (CH_2_) n (42). Reprograming of lipid metabolism of tumor cells may promote cell migration (47, 48), suggesting that mitochondrial fatty acid metabolism could serve as the energy base for regulating migration processes that are important for metastasis.

**Figure 9:**
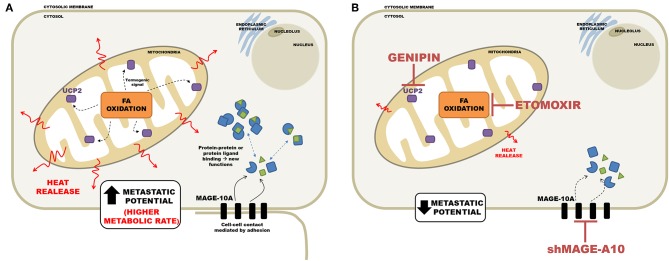
Schematic representation of the relationship between metastatic potential and heat release in the tumor cell. **(A)** Cell with high metastatic potential has important proteins for regulating functioning cell-cell interaction and migration, such as MAGEA10; as well as an active mitochondria, the communication between β-oxidation and high levels of UCP2, resulting in a high thermogenesis. **(B)** Silencing of MAGEA10 and/or pharmacological inhibition of UCP2 and CPT1-A, results in less heat release. This suggests an important participation of these proteins in thermogenesis.

In conclusion the diagram in Figure 9 summarizes the main findings of this work and shows the main regulatory events in metastasis. The isothermal titration microcalorimetry approach used in the present work afforded a non-invasive, real-time, sensitive way to assess the net energy output of living tumor cells. We argue that the data presented here reflected mainly the summation of enthalpies related to metabolic rates and that mitochondrial metabolism may occupy a central role in sustaining the malignant phenotype.

## Data Availability Statement

The datasets generated for this study are available on request to the corresponding author.

## Author Contributions

DL, TO, LM, and VA performed the experiments. MR contributed to cell culture. LK supervised the microcalorimetry experiments. FR conceived the experiments, coordinated the project, and wrote the manuscript.

### Conflict of Interest

The authors declare that the research was conducted in the absence of any commercial or financial relationships that could be construed as a potential conflict of interest.

## Abbreviations

ATP: adenosine triphosphate
BSA: bovine serum albumin
CPT-1: carnitine palmitoyl transferase-1
DMEM: Dulbecco minimal essential medium
DTT: dithiothreitol
EDTA: ethylene diamine tetra acetate
FBS: fetal bovine serum
ITC: isothermal titration calorimetry
LDH: lactate dehydrogenase
LN: lymphonode
MAGEA10: Melanoma associated gene 10
MTT: 3-[4,5- dimethylthiazole-2-yl]-2,5-diphenyltetrazolium bromide
OXPHOS: oxidative phosphorylation
PCR: polymerase chain reaction
SCC: squamous carcinoma cells
UCP2: uncoupling protein 2.
