# Corrigendum: Isothermal Microcalorimetry of Tumor Cells: Enhanced Thermogenesis by Metastatic Cells

**DOI:** 10.3389/fonc.2020.00367

**Published:** 2020-03-24

**Authors:** Douglas Lemos, Thaís Oliveira, Larissa Martins, Vitória Ramos de Azevedo, Mariana Figueiredo Rodrigues, Luisa Andrea Ketzer, Franklin David Rumjanek

**Affiliations:** ^1^Laboratório de Bioquímica e Biologia Molecular Do Câncer, Instituto de Bioquímica Médica Leopoldo de Meis, Universidade Federal Do Rio de Janeiro, Rio de Janeiro, Brazil; ^2^Núcleo Multidisciplinar de Pesquisa UFRJ-Xerém em Biologia (NUMPEX-Bio), Universidade Federal Do Rio de Janeiro, Duque de Caxias, Brazil

**Keywords:** thermogenesis, microcalorimetry, metastasis, UCP2, etomoxir, fatty acid oxidation

In the original article, there was a mistake in Figure 8 as published. Figure 5 and 8 were erroneously duplicated. Both figures correspond to the effect of genipin on heat release. Figure 8 should correspond to the effect of etomoxir. The corrected Figure 8 appears below.

**Figure 8:**
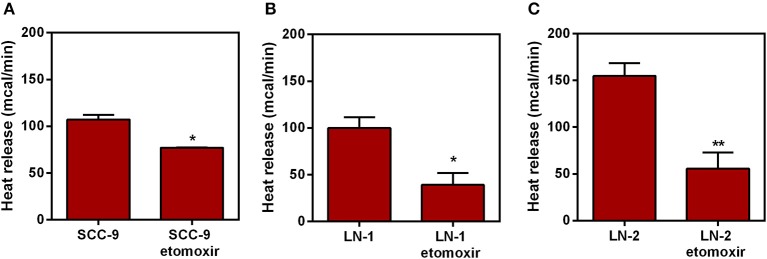
Effect of etomoxir on the heat release by human oral squamous carcinoma cells SCC-9, LN-1 and LN-2 cells. The bars represent the release of total heat of living cells in 35 min of experiment. **(A)** Heat release by SCC-9 cells untreated and treated with 300 μM of etomoxir; **(B)** Heat release by LN-1 cells untreated and treated with 300 μM of etomoxir. **(C)** Heat release by LN-2 cells untreated and treated with 300 μM of etomoxir. Values were expressed as mean ± SEM. *p < 0.05; **p < 0.01.

The authors apologize for this error and state that this does not change the scientific conclusions of the article in any way. The original article has been updated.

