# Supplementary material for: Isothermal Microcalorimetry of Tumor Cells: Enhanced Thermogenesis by Metastatic Cells
Source: Front Oncol. 2019 Dec 18;9:1430. doi: 10.3389/fonc.2019.01430 (PMC6930183; doi:10.3389/fonc.2019.01430)
Supplement: Supplementary file 1 [file Data_Sheet_1.docx]

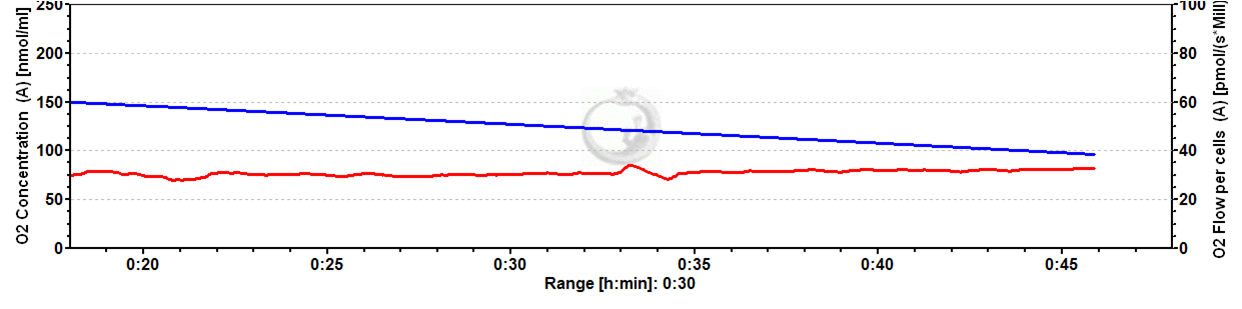


**Supplementary Figure 1.** Representative graph of the oxygen consumption of the cells in 45 minutes. The cells remained in closed chamber under constant agitation for 45 minutes, during which period the cellular oxygen consumption (red line) and the oxygen concentration in the chamber (blue line) were measured.





**Supplementary Figure 2.** The heat released by different types of intact tumor cells is constant. The gradient from gray to red circles represent increasing metastatic potentials. **(A)** Murine melanoma cells 4C, 4C11- and 4C11+; **(B)** human melanoma cells WM983A, WM983B and WM852; **(C)** human non-small-cell lung adenocarcinoma cells A549 and H460; **(D)** human oral squamous carcinoma cells SCC9, LN-1 and LN-2 and **(E)** human breast cancer cells MCF-7 and MDA-MB-231. In the graph is represented the release of total heat of living cells in 35 minutes of experiment. Values ​​were expressed as mean ± SEM.


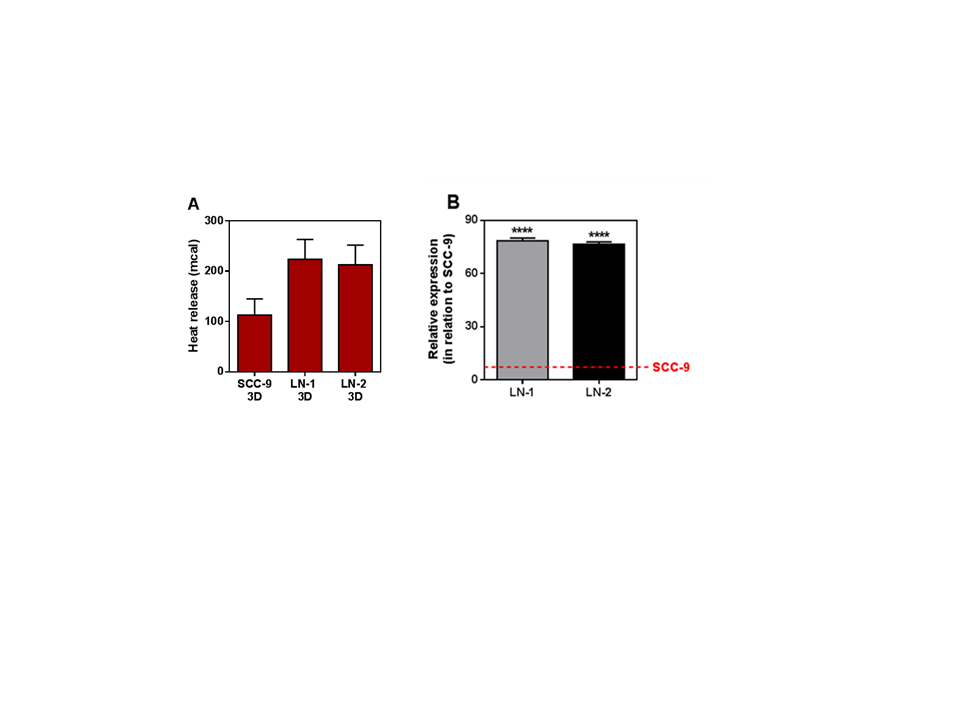


**Supplementary Figure 3.** Heat release and UCP2 mRNA expression by human oral squamous carcinoma cells cultivated in spheroids. **(A)** SCC-9, LN-1 and LN-2 spheroids (3D) were dissociated prior to heat change measurements. The bars represent the release of total heat of living cells in 35 minutes of experiment; **(B)** mRNA expression levels of UCP2 in SCC-9, LN-1 and LN-2 spheroids (3D) SCC-9 (red line), LN-1 (grey bar) and LN-2 (black bar). Values ​​were expressed as mean ± SEM. **** p<0,0001.





**Supplementary Figure 4.**Cell viability of human oral squamous carcinoma cells SCC-9, LN-1 and LN-2 incubated with etomoxir. 2x10^4^ cells per mL were incubated with 50 μM, 100 μM and 300 μM of etomoxir during 35 minutes and the viability of cells was measured by the LDH release **(A)** and MTT assay **(B)** as described in methods. SCC-9 (white bar), LN-1 (grey bar) and LN-2 (black bar). Values ​​were expressed as mean ± SEM.
